# Childhood behaviour problems show the greatest gap between DNA-based and twin heritability

**DOI:** 10.1038/s41398-017-0046-x

**Published:** 2017-12-12

**Authors:** Rosa Cheesman, Saskia Selzam, Angelica Ronald, Philip S. Dale, Tom A. McAdams, Thalia C. Eley, Robert Plomin

**Affiliations:** 1 King’s College London, Social, Genetic and Developmental Psychiatry Centre, Institute of Psychiatry, Psychology and Neuroscience, London, UK; 2 Department of Psychological Sciences, Birkbeck, University of London, London, UK; 3 Department of Speech and Hearing Sciences, University of New Mexico, Albuquerque, NM USA

## Abstract

For most complex traits, DNA-based heritability (‘SNP heritability’) is roughly half that of twin-based heritability. A previous report from the Twins Early Development Study suggested that this heritability gap is much greater for childhood behaviour problems than for other domains. If true, this finding is important because SNP heritability, not twin heritability, is the ceiling for genome-wide association studies. With twice the sample size as the previous report, we estimated SNP heritabilities (*N* up to 4653 unrelated individuals) and compared them with twin heritabilities from the same sample (*N* up to 4724 twin pairs) for diverse domains of childhood behaviour problems as rated by parents, teachers, and children themselves at ages 12 and 16. For 37 behaviour problem measures, the average twin heritability was 0.52, whereas the average SNP heritability was just 0.06. In contrast, results for cognitive and anthropometric traits were more typical (average twin and SNP heritabilities were 0.58 and 0.28, respectively). Future research should continue to investigate the reasons why SNP heritabilities for childhood behaviour problems are so low compared with twin estimates, and find ways to maximise SNP heritability for genome-wide association studies.

## Introduction

Behaviour problems including anxiety, depression, autistic-like traits, hyperactivity and conduct problems are common in childhood, with a cumulative incidence of 12% for one or more disorders^1^. Such problems often persist: children with behaviour problems in childhood have an increased risk of lifetime psychopathology 24 years later^2^, and half of all lifetime cases of diagnosed psychopathology have their onset by age 14^3^. Evidence from many twin, family, and adoption studies points to significant genetic influence, with heritability estimates ranging from ~ 40% for anxiety and depression to >60% for autistic-like traits and hyperactivity^4^. Moreover, longitudinal studies indicate the presence of stable genetic influences on behaviour problems^5–7^.

Genome-wide association (GWA) studies have sought to identify genetic variants responsible for the heritability of childhood behaviour problems but have so far been overwhelmingly unsuccessful^8–14^. The lack of genetic associations adds to the evidence that these traits are shaped by many common genetic variants with small effects, which will require very large GWA samples to find. The success of the recent large Attention-Deficit/Hyperactivity Disorder GWA study marks the start of progress in this direction^15^.

A quantitative genetic method—genome-wide complex trait analysis (GCTA)—offers insight into how much of this ‘missing heritability’ (the gap between twin heritability and significant associations in GWA studies) could theoretically be identified with GWA analyses. GCTA estimates the extent to which measured single-nucleotide polymorphisms (SNPs) contribute to the heritability of a trait. GCTA estimates of genetic influence, known as SNP heritability, will be lower than twin study-based heritability, partly because GCTA detects only the additive effects of causal variants tagged by common SNPs on current DNA arrays used in GWA research, and not non-additive effects or rare variants. These limitations—additive effects and common SNPs—also apply to GWA research. Thus, the current ceiling for GWA analyses is SNP heritability, not twin heritability.

Few studies have estimated SNP heritability for childhood behaviour problems. The first reports, from the Twins Early Development Study (TEDS), found negligible SNP heritability for childhood behaviour problems at age 12 (0% for self-report, 2% for parent reports and 11% for teacher reports), and a mixture of significant and nonsignificant modest estimates for psychotic experiences at age 16, despite substantial twin heritability estimates from the same sample^16,17^. These low SNP heritability estimates could be owing to low power (*N* of 2000–2700). Seven subsequent studies yielded mixed results for several quantitative measures of childhood behaviour problems, although power was also marginal in these studies. Supplementary Table 1 and the accompanying text summarise and discuss these studies in detail.

The present study extends our previous reports, estimating twin and SNP heritabilities with a sample size almost twice as large for 37 measures of childhood behaviour problems as assessed by parents, teachers, and children themselves at age 16 as well as age 12, as well as cognitive and anthropometric measures for comparison. We now have twice the power to detect low SNP heritability: 70% rather than 35% power to detect a SNP heritability of 20%. We compare SNP heritability estimates directly to heritability estimates derived from twin data using the same sample and the same measures, to investigate the genetic architecture of childhood behaviour problems, and to quantify the likelihood that further GWA studies will find variants involved in their aetiology.

## Materials and methods

### Sample

The sample is drawn from the Twins Early Development Study (TEDS), a multivariate, longitudinal study of >10,000 twin pairs representative of England and Wales, recruited between 1994 and 1996^18^. GCTA analyses for this study were conducted on a sub-sample of unrelated individuals (only one member of each twin pair) with available genome-wide genotyping and behaviour problem data at ages 12 and 16. For the twin modelling, we added the second twin from each pair for individuals included in GCTA, allowing the two heritability estimates to be compared.

**Fig. 1:**
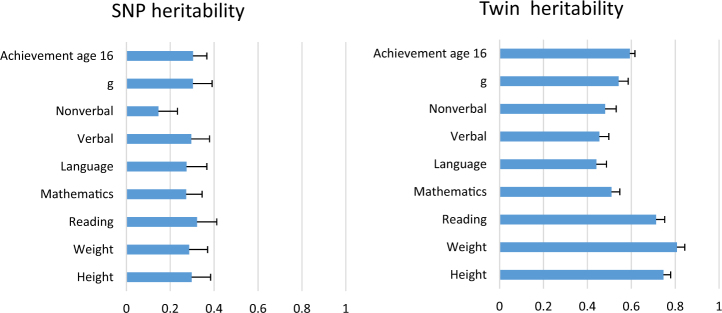
Shows heritability estimates from SNP and twin heritability analyses for cognitive and anthropomorphic measures at 12, plus GCSE achievement at age 16 For the 12-year measures, the GCTA sample size ranged from 3219 for language ability to 3992 for the maths web test. The GCTA sample size for GCSE achievement was 4653. For the twin analyses of 12-year data, the number of twin pairs ranged from 3253 for reading ability to 3994 for maths ability. The sample size for GCSE achievement was 4724 pairs. As for the behaviour problem measures, the sample sizes were lower for GCTA than twin analyses because GCTA removes individuals who are closely related (in the present study IBD > 0.025). Note ‘*g*’ = general intelligence composite. 95% confidence intervals from OpenMx output were converted into standard errors, which are represented by the error bars

**Fig. 2:**
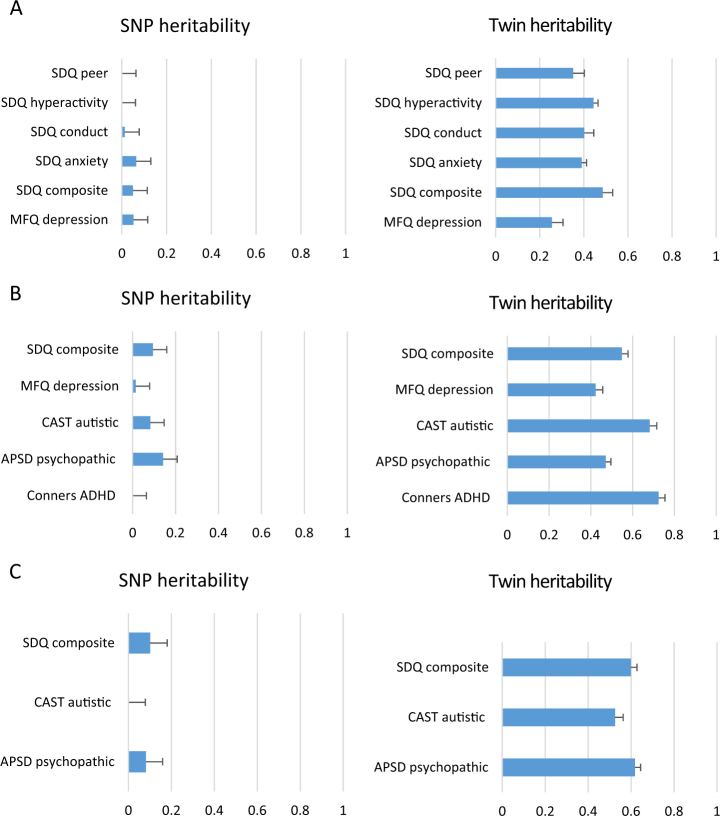
Compares SNP and twin heritability estimates for key composite childhood behaviour problem measures at age 12 for three different reporters (**a**) Self-report (*N* = 4315–4388 for GCTA; *N* = 4393–4467 for twin analyses). (**b**) Parent report (*N* = 4280–4432 for GCTA; *N* = 4367–4502 for twin analyses). (**c**) Teacher report (*N* = 3553–3600 for GCTA; *N* = 3599–3641 for twin analyses)

**Fig. 3:**
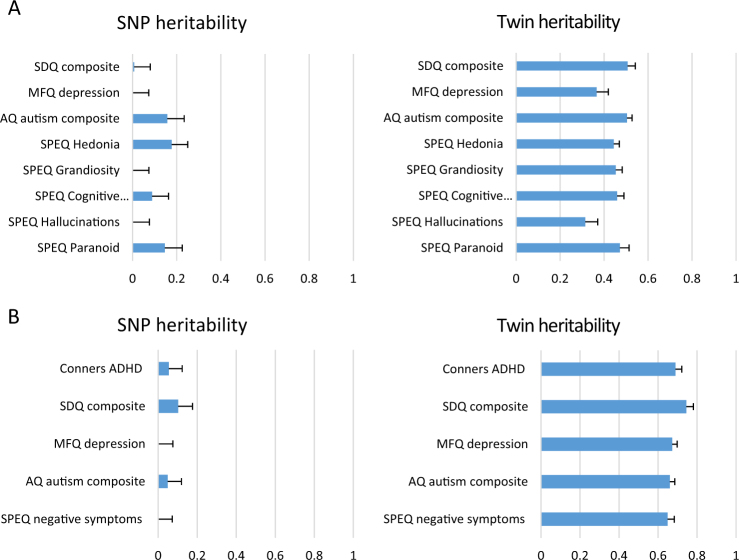
Compares SNP and twin heritability estimates for key composite childhood behaviour problem measures at age 16 for two different reporters (**a**) Self-report (*N* = 3688–3760 for GCTA; *N* = 3745–3823 for twin analyses). (**b**) Parent report (*N* = 3652–3759 for GCTA; *N* = 3716–3820 for twin analyses)

For GCTA analyses, the numbers of individuals with behaviour problem data range from 3553 to 4432 at age 12, and from 3652 to 3760 at 16. For twin analyses, the numbers of twin pairs with behaviour problem data at age 12 range from 3599 to 4467 and from 3716 to 3823 at 16. See legends of Figures 1–3 (in Results) for more details of sample sizes.

As previously mentioned, with our greater sample size (average *N* = 3927, rather than 2486), we have approximately twice the power of the previous TEDS studies. If, as with our cognitive and anthropometric traits, true SNP heritability of behaviour problems is half that of our average twin estimate (52%), we have 90% power (up to 95% with maximum *N* = 4432) rather than 53% as with the previous sample.

### Genotyping

DNA from 3665 individuals extracted from buccal cheek swabs was hybridised to AffymetrixGeneChip 6.0 SNP genotyping arrays using standard WTCCC2 experimental protocols. In total, 4649 other individuals of European ancestry were genotyped on HumanOmniExpressExome-8v1.2 arrays at the Molecular Genetics Laboratories of the Medical Research Council Social, Genetic Developmental Psychiatry Centre, based on DNA extracted from saliva samples.

After initial quality control and genotype calling, the same quality control was conducted on the samples genotyped on the Affymetrix and Illumina platforms separately using PLINK^19,20^, R^21^, and vcftools^22^. Samples were removed based on call rate (<0.99), suspected non-European ancestry, heterozygosity, array signal intensity, and relatedness. SNPs were excluded if minor allele frequency was <0.5%, if >1% of genotype data were missing, or if the Hardy Weinberg p-value was lower than 10^−5^. Non-autosomal markers and indels were removed. Associations between the SNP and the platform, batch, or plate on which samples were genotyped were calculated; SNPs with *P*-values <10^−3^ were excluded. A total sample of 6710 individuals remained after quality control—3093 individuals and 525,859 SNPs genotyped on the Affymetrix platform and 3617 individuals and 600,034 SNPs genotyped on the Illumina platform.

Genotypes from the two platforms were separately imputed using the Haplotype Reference Consortium 5 and Minimac3 1.0.13^23,24^ before merging genotype data obtained from both arrays.

We performed principal component analysis on a subset of 42,859 common (MAF > 5%) autosomal HapMap3 SNPs^25^, after stringent pruning to remove markers in linkage disequilibrium (*r* ^2^ > 0.1) and excluding high linkage disequilibrium genomic regions so as to ensure that only genome-wide effects were detected.

For twin analyses, DZ same-sex and opposite-sex pairs were combined to increase power and because previous sex-limitation analyses of TEDS data show zero or inconsistent evidence of qualitative or quantitative sex differences^26^, as confirmed in this study (see Supplementary Tables 2 and 3). Moreover, splitting the GCTA sample by sex would dramatically decrease power, preventing the comparison of twin and SNP heritability estimates, which is the focus of this paper.

### Measures

Twins Early Development Study (TEDS) measures have been described previously. In this study, we examined the same measures as in the previous publications^16, 17^, with the addition of various self- and parent-report measures of behaviour problems at 16 and educational achievement at 16.

For all phenotypes, z-standardised residuals were derived for each scale regressed on sex and age (since age and sex are perfectly correlated across pairs, this would be misinterpreted as shared environment in twin analyses). Outliers above or below 3 standard deviations from the mean were excluded and the scale was quantile normalised (with van der Waerden transformation) before analysis^27^. Composite scores were calculated as unit-weighted means, given complete data for more than half the measures contributing to the composite (3 of 4 or 2 of 3 sub-scales). All procedures were executed using R. These data preparation steps are the same as the previous study^16^ to enable comparison.

#### 12 –year measures

The Moods and Feelings Questionnaire (MFQ) (self- and parent-rated) was used to assess depressive symptoms.

The Strengths and Difficulties Questionnaire (self-, parent-, teacher-rated) included four scales for anxiety, conduct, hyperactivity and peer problems, from which a mean composite was created.

The Antisocial Process Screening Device (APSD) (parent-rated) was measures three types of psychopathic traits: callous-unemotional, impulsivity, and narcissism.

The Childhood Autism Spectrum Test (CAST) (parent-rated) assessed children’s autistic-like traits overall and for three subscales: communication, social and non-social.

Conners’ (parent- and teacher-rated) scales measured ADHD behaviours for the subscales of inattention and hyperactivity-impulsivity.

Reading ability was measured using the reading comprehension subtest of the Peabody Individual Achievement Test (PIAT), the Global Online Assessment for Learning (GOAL) Formative Assessment in Literacy for Key Stage 3, an adaptation of the Woodcock-Johnson III Reading Fluency Test (WJRF) and by the Test of Word Reading Efficiency (TOWRE).

Mathematics ability was assessed with three scales based on the National Foundation of Educational Research 5–14 Mathematics Series: understanding numbers, non-numerical processes, and computation and knowledge.

Language ability scales covered syntax (Listening Grammar subtest of the Test of Adolescent and Adult Language), semantics (Level 2 of the Figurative Language subtest of the Test of Language Competence), and pragmatics (Level 2 of the Making Inferences subtest of the Test of Language Competence).

Verbal ability was tested with the Wechsler Intelligence Scale for Children as a Processing Instrument (WISC-III-PI) Multiple Choice Information (General Knowledge) and Vocabulary Multiple Choice subtests. Nonverbal reasoning tests were Wechsler Intelligence Scale for Children–III (WISC-III-UK) Picture Completion and Raven’s Standard and Advanced Progressive Matrices. General cognitive ability was indexed as a composite of the four verbal and nonverbal tests.

Height and weight were self-reported at age 12.

#### 16-year measures

As at age 12, the MFQ (self- and parent-rated) was used to assess depressive symptoms; Conners’ parent-rated questionnaire measured ADHD behaviours for the subscales of inattention, impulsivity, and emotional lability; and the SDQ (self- and parent-rated) measured anxiety, conduct, hyperactivity and peer problems, although parent-rated anxiety was not collected at age 16.

In addition, the Autism Quotient scale (self- and parent- report) measured autistic traits, including subscales for attention to detail and social skills.

The Specific Psychotic Experiences Questionnaire (SPEQ) (self-reported) assessed individual psychotic experiences^28^. The measure consists of five self-report subscales, which are always analysed individually: Paranoia, Hallucinations, Cognitive Disorganisation, Grandiosity, and Hedonia, and a parent-report scale of Negative Symptoms.

Achievement was reported as a mean GCSE grade for the core subjects of English, Mathematics, and Science.

### Statistical analyses

Genomic relatedness matrix restricted maximum likelihood (GREML), implemented in the Genome-wide Complex Trait Analysis (GCTA) programme, estimates genetic influence directly using individual-level genome-wide genotypes in large samples of unrelated individuals^29^. The first step in GCTA is the calculation of genetic similarity for each pair of unrelated individuals across all genotyped or imputed genetic markers. Each pair’s genetic similarity is then used to predict their phenotypic similarity. In the present study, individuals with pairwise identity-by-descent (IBD) of >0.025 were removed, so that chance genetic similarity can be used as a random effect in a linear mixed model. By comparing a matrix of pairwise genomic similarity to a matrix of pairwise phenotypic similarity using a random-effects mixed linear model, the variance of a trait can be decomposed into genetic and residual components, using residual maximum likelihood. GCTA detects only those genetic effects tagged by the common SNPs (in this study allele frequencies > 5%) that are included in commercially available DNA arrays used in GWA studies; the residual component includes any source of variance that is not explained by additive effects of common SNPs, including non-additive genetic effects, rare variants, environment, gene–environment interaction, and error. In our GCTA analyses, phenotypic data were prepared as detailed above, and we used the first 10 principal components as covariates.

Twin maximum-likelihood model fitting using full-information matrices was carried out using structural equation modelling software OpenMx in R^30^. Standard errors were derived from 95% confidence intervals. We used the full ACE model (decomposing variance into additive genetic, shared environmental and non-shared environmental components respectively) to enable comparison with GCTA results, even when C was not significant (dropping C can inflate A). We fit ADE models when the DZ correlation was less than half the MZ correlation. This correlation pattern indicates non-additivity because gene-gene interactions are shared by MZ twins but not necessarily by DZ twins, therefore contributing to lower similarity between DZ twins.

The differences between genetic and environmental variance components estimated by twin and GCTA analyses should be noted. ‘A’ in twin models includes additive genetic effects of any DNA sequence differences, and is distinguished from ‘C’ as well as ‘E’. In contrast, the equivalent of ‘A’ from GCTA includes only common SNPs in current DNA arrays, and other genetic variance not explained by these SNPs loads onto the environmental component, which is not further decomposed into shared and non-shared environment, and includes non-additive and rare variant effects. A further difference is that standard errors are larger for SNP heritability than for twin estimates because GCTA is based on comparisons of small pairwise genetic differences (IBD < 0.025) compared to the genetic difference of 100 vs. 50% for MZ and DZ twins.

Sensitivity analyses were performed to examine the influence of the van der Waerden quantile normalisation on heritability estimates for the behaviour problem phenotypes, which are highly skewed, and to examine the effects of treating outliers in different ways. It has been suggested that removal of extreme cases decreases SNP heritability because these individuals might be genuine, biologically significant outliers^31^. First, we carried out analyses in the same way except without performing the quantile normalisation. Second, we compared the original analyses to SNP heritability estimates when outliers were kept in. Third, we kept the analyses the same but winzorised outliers rather than removing them, by creating z-scores and then fixing the outliers at z-score 3.29 (the cut-off for 0.1% of scores).

## Results

Figure 1 shows SNP and twin heritabilities for height, weight, cognitive abilities, and educational achievement. Twin heritability estimates are ~ 80% for height and weight, ~ 50% for cognitive traits, and ~ 60% for achievement. SNP heritability estimates are ~ 30% for height and weight, ~ 25%, for the cognitive traits, and 30% for achievement at 16. As expected given the limitations of estimating heritability using common SNPs, SNP heritability estimates are around half the twin estimates.

Figure 2 (age 12) and Figure 3 (age 16) show a very different pattern of SNP and twin heritability estimates for behaviour problems. The figures include a subset of our measures that cover the breadth of behaviour problem traits. SNP heritabilities for behaviour problems are much lower than half the twin estimates. Full SNP and twin heritability results for all 37 measures with sample sizes and standard errors are included in Supplementary Table 4. Average twin heritabilities are 37% for self-report (18 measures), 60% for parent ratings (21 measures), and 58% for teacher ratings (three measures). In contrast, for these same measures, average SNP heritabilities are 5% for self-report, 7% for parent ratings, and 6% for teacher ratings. The average percentage of twin heritability accounted for by SNP heritability is roughly equal across these three categories: 13.5%, 11.7%, and 10.3%. For the 37 traits in Supplementary Table 4, only five show significant SNP heritability.

Sensitivity analyses were performed to examine the influence of quantile normalisation and removal of outliers on heritability estimates. Results do not differ with and without transformation, as indicated by overlapping standard errors and similar point estimates (never a difference of >0.04). In additional sensitivity analyses we performed quantile normalisation, but then varied the treatment of outliers. There was no difference in estimates associated with removing, retaining or winzorising outliers (see Supplementary Figures 1–5 for plots of the sensitivity analyses).

Because non-additive genetic variance could lower SNP heritability estimates, we tested for non-additive genetic influence in twin analyses by fitting ADE models and comparing the fit with the ACE models. Lower AIC (Akaike’s Information Criterion) indicates better fit to the data. ADE models were not significantly better fitting for height, weight, cognitive abilities and educational achievement. However, ADE models fit significantly better for seven of the 37 behaviour problem variables, although confidence intervals included zero for four of these (see Supplementary Table 5).

## Discussion

We estimated twin and SNP heritabilities for diverse domains of childhood behaviour problems as rated by parents, teachers, and children themselves at ages 12 and 16 in a large UK-representative sample. We found surprisingly low SNP heritability estimates (average 6%) in comparison to twin heritabilities (average 52%). This is particularly striking given that SNP-based estimates were half those of twin-based estimates for anthropomorphic and cognitive measures. Although we found significant SNP heritability for a few behaviour problems, it is more appropriate to emphasise the average SNP heritability estimates for the whole study rather than findings for a few measures that might be due to chance. Our results are strengthened by sensitivity analyses indicating that low SNP heritability estimates are not due to approaches taken to outliers or normalisation of the positively skewed behaviour problem distributions.

These results thus confirm and extend our previous report of a particularly large gap between SNP heritability and twin heritability for quantitative measures of behaviour problems in children. As detailed in Supplementary Table 1, previous studies in other samples have yielded somewhat higher SNP heritability estimates for several quantitative measures of childhood behaviour problems. However, these results have been very mixed and their power has been marginal. This contrasts with the consistency of our finding that SNP heritability estimates are considerably less than half the size of twin heritability estimates across a range of domains of behaviour problems and across self-, parent- and teacher ratings. Furthermore, our twin heritability estimates for both behaviour problems and cognitive abilities are similar to those reported in the literature, suggesting that our SNP heritability results are not attributable to measurement issues specific to behaviour problems in this sample. Finally, our SNP heritability estimates for cognitive abilities are similar to those previously reported—about half the twin study heritability estimates. This demonstrates that our low SNP heritability estimates for behaviour problems are specific to behaviour problems and not due to methodological problems with estimating SNP heritability.

We have limited our discussion thus far to studies of quantitative measures of behaviour problems in children (in Supplementary Table 1), to enable direct comparison to our sample. However, quantitative measures of behaviour problems in adults also yield results similar to ours. For example, in studies of quantitative measures of anxiety, SNP heritability was estimated as 5% in a sample of 10,414^32^ and 10% in a sample of 5362^33^. Several studies of adults have estimated SNP heritability for neuroticism, a trait that is associated with negative emotional states and psychiatric disorders^34^ and has a strong positive genetic correlation with depression (0.83)^35^. Despite substantial twin heritability (~ 40%)^36^, SNP heritability of neuroticism is low: 6% (*N* = ~ 12,000)^37^ 10% (*N* = ~ 10,000)^38^ and 13% in UK Biobank (N > 100,000)^39^. Low SNP heritability has also been reported for Linkage Disequilibrium Score Regression analysis (LDSR), which uses GWA summary statistics to estimate SNP heritability. For example, LDSR estimates of SNP heritabilities for quantitative measures of depression and neuroticism in a sample of >160,000 individuals are just 0.05 and 0.09^40^.

Results from case-control studies are more difficult to compare to our results because case-control studies rely on the hypothetical construct of a continuous liability but the analyses are based on a dichotomous trait. SNP heritability of ADHD and ASD is substantial in case-control studies (0.20–0.30)^41,42^, yet still less than half of the twin heritability. A large UK Biobank study (N > 100,000) found SNP heritability of ~ 10% for binary self-report depression items^39^.

If SNP heritability of childhood behaviour problems is truly as low as 6%, genome-wide association (GWA) studies will struggle to find variants involved in their aetiology, because both GWA and SNP heritability are limited to additive effects of common variants.

One way to improve this situation is to investigate why SNP heritability for behaviour problems in childhood is so low compared to twin estimates. Gene-gene interactions, gene-by-shared-environment interactions, and rare alleles are captured in twin but not SNP heritability estimates, which only establishes how much variance can be explained by additive genetic effects tagged by common SNPs on genotyping arrays. Methods exist to investigate the relationship between genetic architecture and SNP heritability. For example, GREML-MS stratifies SNPs by minor allele frequency (MAF) to allow the data to reveal how much variance is explained by each MAF bin^43^. Models that take MAF and linkage disequilibrium into account have obtained higher estimates of SNP heritability^44^. However, there is little evidence that these factors are more prominent for childhood behaviour problems than other domains, so they seem unlikely to explain the gaping gap between SNP and twin heritability specifically for these traits.

We found some evidence for non-additive genetic influence on several behaviour problem phenotypes in our sample, particularly for hyperactivity and autism, as we would expect from the literature. In contrast, for the cognitive and anthropometric phenotypes, there was no indication of non-additivity. As suggested in the previous report from TEDS, non-additive genetic effects are potentially important in the aetiology of childhood behaviour problems^16^. However, four of the seven traits for which an ADE model was better-fitting also showed significant SNP heritability (self-reported autism, hedonia, cognitive disorganisation, and paranoia at age 16). This pattern is the opposite of what might be expected if dominance were deflating SNP heritability, which includes only additive effects. Moreover, if non-additive genetic effects are responsible for the gap between SNP and twin heritability estimates for behaviour problems, it will be difficult for GWA studies to achieve adequate power to detect them^45^.

A more hopeful possibility lies with novel approaches to the study of childhood behaviour problems that increase SNP heritability. Because we used the same measures to assess SNP heritability and twin heritability, improved measurement of behaviour problems is unlikely to narrow the gap between these heritability estimates. However, anything that increases SNP heritability for behaviour problems could be helpful in relation to future GWA studies.

Three examples of this approach that each involve multivariate genetic analyses could lead to increased SNP heritability by creating genetically sensitive composites of behaviour problems across raters, across time and across traits. With respect to raters, self-report, parent ratings, and teacher ratings of behaviour problems correlate only modestly phenotypically (~0.30)^46–50^. Multivariate genetic analyses indicate higher genetic correlations across raters, suggesting that what the raters see in common about children’s behaviour problems captures more of the genetic action, although there are also rater-specific genetic influences^47, 51^. Thus, one possibility for increasing SNP heritability is to create cross-rater composites that reflect their genetic correlations, for example, creating a higher-order factor comprising only common variance.

The second example takes advantage of the fact that longitudinal stability of behaviour problems is moderate and mostly attributable to genetic factors^7^. This finding suggests that a cross-age composite guided by age-to-age genetic correlations might yield higher SNP heritability than any measure at a single age. Again, the idea is to establish measures with higher heritability—not only to increase reliability and therefore the proportion of genetic relative to environmental (including error) variance. The third example is that multivariate genetic analyses suggest substantial genetic overlap across some behaviour problem domains^52^. A measure that captures more of the common genetic variance might show higher SNP heritability than most of its constituent sub-scales, as previously demonstrated for the ‘general psychopathology factor’ in childhood^53^.

We are currently testing these hypotheses in TEDS by conducting multivariate genetic analyses across raters, across age, and across domains. We will create behaviour problem composite measures guided by these multivariate genetic results and test the hypothesis that these composites will yield higher SNP heritabilities.

Another approach for increasing heritability is to use item response theory to construct more reliable behaviour problem phenotypes and decompose phenotypic variance into genetic and environmental components^54^. Advanced phenotypic modelling should include testing the unidimensionality, measurement invariance, difficulty, and discrimination (resolution of phenotypic differences between individuals) of our items and measures^55,56^.

In conclusion, we have confirmed a substantial gap between twin and SNP heritabilities of diverse domains of childhood behaviour problems as rated by parents, teachers, and children themselves at ages 12 and 16 in our sample. Future research should continue to investigate the reasons why SNP heritabilities for childhood behaviour problems are so low compared with twin estimates, and pursue methods that maximise SNP heritability for GWA studies.

## Electronic supplementary material

Supplementary Information
