# Supplementary material for: Childhood behaviour problems show the greatest gap between DNA-based and twin heritability
Source: Transl Psychiatry. 2017 Dec 12;7:1284. doi: 10.1038/s41398-017-0046-x (PMC5802501; doi:10.1038/s41398-017-0046-x)
Supplement: Supplementary file 1 — Supplementary Information [file 41398_2017_46_MOESM1_ESM.docx]

**Supplementary information**

**Contents:**

Literature review

Supplementary Table 1: literature review of SNP heritability estimates of quantitative measures of behaviour problems in other samples, with accompanying text discussing the studies.

Full results of present study

Supplementary Tables 2 & 3: sex limitation model-fitting sub-model comparison and full results, respectively.

Supplementary Table 4: full SNP and twin heritability results for the present study.

Supplementary Figures 1-5: results of sensitivity analyses comparing different treatment of outliers and skewness on SNP heritability estimates.

Supplementary Table 5: ADE twin model fitting results.

References.

Literature review

**Supplementary Table 1.** Note: * = standard error was not reported by the authors.

| **Reference** | **Year** | **Trait** | **Sample** | **Measure** | **Age** | **Reporter** | **N** | **SNP heritability** | **SE** |
| --- | --- | --- | --- | --- | --- | --- | --- | --- | --- |
| Benke et al. | 2014 | Internalising | Gen R | CBCL 1.5-5 internalising scale | 3 | Primary caregiver | 2037 | 0.26 | * |
|  |  |  | NTR | CBCL 2-3 internalising scale | 3 | Mother | 1475 | 0.18 | * |
|  |  |  | Raine | CBCL 2-3 internalising scale | 2 | Primary caregiver | 1084 | 0.13 | * |
| Middeldorp et al. | 2016 | ADHD symptoms | ALSPAC | SDQ hyperactivity | 4 | Mother | 5510 | 0.05 | 0.06 |
|  |  |  | ALSPAC | SDQ hyperactivity | 9 | Mother | 5303 | 0.14 | 0.07 |
|  |  |  | NTR / Gen R | CBCL attention problems | 3 & 6 | Mother | 2958 | 0.13 | 0.11 |
|  |  |  | NTR / Gen R | TRF | 7 | Teacher | 1901 | 0.34 | 0.17 |
| Neumann et al. | 2016 | General Psychopathology factor | Gen R | Includes CBCL, Social Responsiveness Scale, CPRS-R, TRF, Berkeley Puppet Interview |  | Primary caregiver and teacher | 2115 | 0.38 | 0.16 |
| Pappa et al. | 2015 | Internalising | NTR / Gen R | CBCL internalising | 7 | Parent | 3175 | 0.12 | 0.10 |
|  |  | Externalising |  | CBCL externalising | 7 | Parent | 3174 | 0.12 | 0.10 |
|  |  | Total behaviour problems |  | CBCL | 7 | Parent | 3175 | 0.18 | 0.10 |
|  |  | Pervasive developmental disorder |  | CBCL PDD | 3 | Parent | 3015 | 0.16 | 0.11 |
|  |  | ADHD symptoms |  | CPRS ADHD combined scale | 8 | Parent | 2262 | 0.40 | 0.14 |
|  |  | ADHD symptoms |  | CPRS ADHD inattentive | 8 | Parent | 2264 | 0.37 | 0.14 |
|  |  | ADHD symptoms |  | CPRS ADHD hyperactive-impulsive | 8 | Parent | 2260 | 0.45 | 0.14 |
|  |  | Oppositional-defiant disorder |  | CPRS ODD scale | 8 | Parent | 2262 | 0.20 | 0.14 |
|  |  | Attention problems |  | TRF Attention problems | 7 | Teacher | 1495 | 0.71 | 0.22 |
|  |  | Externalising |  | TRF Externalising | 7 | Teacher | 1495 | 0.44 | 0.22 |
| Sallis et al. | 2017 | Depressive Symptoms | ALSPAC | Short Moods and Feelings Questionnaire (SMFQ) | 11 | Child | 5480 | 0.07 | 0.06 |
|  |  |  |  |  | 13 | Child | 5055 | 0.17 | 0.06 |
|  |  |  |  |  | 14 | Child | 4615 | 0.02 | 0.07 |
|  |  |  |  |  | 17 | Child | 3605 | 0.08 | 0.09 |
|  |  |  |  |  | 18 | Child | 3289 | 0.03 | 0.10 |
|  |  |  |  |  | 19 | Child | 2433 | 0.05 | 0.13 |
|  |  |  |  |  | 10 | Parent | 5571 | 0.04 | 0.06 |
|  |  |  |  |  | 12 | Parent | 5119 | 0.12 | 0.06 |
|  |  |  |  |  | 13 | Parent | 5031 | 0.07 | 0.07 |
|  |  |  |  |  | 17 | Parent | 3990 | 0.04 | 0.08 |
| St Pourcain et al. | 2014 | Social-communication spectrum | ALSPAC | SCDC | 8 | Mother | 5204 | 0.24 | 0.07 |
|  |  |  |  |  | 11 | Mother | 5121 | 0.16 | 0.07 |
|  |  |  |  |  | 14 | Mother | 4797 | 0.08 | 0.07 |
|  |  |  |  |  | 17 | Mother | 4026 | 0.45 | 0.08 |
| Stergiakouli et al. | 2017 | Social-communication spectrum | ALSPAC | SCDC | 8 | Mother | 5136 | 0.24 | 0.07 |
|  |  | Social-communication spectrum | ALSPAC | SCDC | 11 | Mother | 5056 | 0.16 | 0.07 |
|  |  | Social-communication spectrum | ALSPAC | SCDC | 14 | Mother | 4735 | 0.08 | 0.07 |
|  |  | ADHD symptoms | ALSPAC | SDQ-ADHD | 7 | Mother | 5185 | 0.10 | 0.07 |
|  |  | ADHD symptoms | ALSPAC | SDQ-ADHD | 10 | Mother | 5235 | 0.14 | 0.07 |
|  |  | ADHD symptoms | ALSPAC | SDQ-ADHD | 12 | Mother | 4886 | 0.19 | 0.07 |
|  |  | ADHD symptoms | ALSPAC | SDQ-ADHD | 13 | Mother | 4735 | 0.18 | 0.07 |
|  |  | Social-communication spectrum | ALSPAC | SCDC | 17 | Mother | 3977 | 0.45 | 0.09 |
|  |  | ADHD symptoms | ALSPAC | SDQ-ADHD | 17 | Mother | 3968 | 0.15 | 0.09 |

We know of seven studies in other samples that have estimated SNP heritabilities for quantitative measures of childhood behaviour problems (see Supplementary Table 1). The studies all come from the UK Avon Longitudinal Study of Parents and Children (ALSPAC) sample, the Netherland Twin Register (Twin Study), or the Rotterdam Netherlands Generation R study, so there is some overlap in participants between some studies.

From these seven studies, average SNP heritability estimates are 19% (average N =1532) (1), 16% (average N = 3918) (2), 38% (N = 2115) (3), 35% (average N =2298) (4), 7% (average N =4419) (5), 23% (average N = 4787) (6), and 19% (average N =4768) (7) respectively. The average SNP heritability across these seven studies is 20%. To allow larger studies to count for more towards the average, we calculated average SNP heritability of the seven studies weighted by N. Multiplying each average SNP heritability by the average sample size for each study, summing these, and then dividing by total average sample size, the figure was still 20%.

The SNP heritability estimates are high, but are difficult to interpret because the range is large (2% to 71%) and power was generally marginal. For example, the study with the highest average SNP heritability produced estimates varying from 12 to 71% (4). The maximum of 71% was found for attention problems at age 7, with a standard error of 0.22. The four sub-scales with better power than the others (standard errors of 0.10 and 0.11) yielded much lower SNP heritability (14% rather than 34% on average). Interestingly, composites showed higher SNP heritability than individual scales: 0.18 for total CBCL score (4) and 0.38 for the ‘general psychopathology factor’ (3), although standard errors were high (0.10 and 0.16 respectively).

Papers using the ALSPAC sample estimated substantial additive genetic heritability for social communication difficulties at ages 8 and 17 (0.24 and 0.45 respectively), and with sample sizes of ~4000-5000, power was reasonably high (7). However, these results are difficult to interpret because SNP heritability was significantly lower at ages 11 and 14 (0.16 and 0.08 respectively). In the same sample, ADHD symptoms showed the reverse developmental pattern, with SNP heritability increasing from age 7 to 12 and then decreasing by age 17 (7). Similarly, one study reported the SNP heritability of ADHD symptoms to be as high as 0.34 at age 7, but the standard error was large (0.17) and SNP heritability was only 0.05 at age 4 with a larger sample and smaller standard error (0.06) (2).

Out of the papers discussed here, those using ALSPAC data are the only ones with substantial power (SE <0.10), and the average SNP heritability of autism and ADHD related traits in these studies was 0.19, which is less than half of twin heritability estimates for these traits (>60%). Notably, SNP heritability was very low for both self- and parent-rated depressive symptoms in ALSPAC (N=~4500), with standard errors crossing zero for all the estimates apart from self-rated depressive symptoms at age 13 (5).

The rest of the studies in the literature review estimating SNP heritabilities for internalising traits did not have adequate power to detect SNP heritability of half the twin estimates (30-40%): over 5000 people are needed for at least 66% power to detect SNP heritability of 15%.

TEDS studies published in 2013 using the smaller sample that were originally genotyped found consistently low SNP heritability (relative to twin heritability) for childhood behaviour problems (8–11). The average SNP heritability across all ten studies (including TEDS studies) is 14% (range 0-71%). The average SNP heritability of the 10 studies weighted by N is 19%.

Aside from the problem of power, why do we find such mixed results for the SNP heritabilities of childhood behaviour problems, with TEDS estimates being lower than others? The sensitivity analyses performed in this study (see Supplementary Figures 1-5) demonstrates that treatment of outliers and distributional skew likely does not affect SNP heritability estimates, and it is unlikely that differences in ancestry explain the mixed results given that ALSPAC (UK-based) estimates were more similar to those from the Netherlands than to TEDS.

Further clarifying this inconsistent and underpowered literature led us conduct the present study. We suggest that the mixed results remain a puzzle: variation in estimates is wide, as are standard errors, and the field requires further analyses using larger samples across the life-span to clarify the precisely how much variation in behaviour problems phenotypes can be accounted for by common variants. However, TEDS findings are strengthened by the richness of the study’s longitudinal data – for example, by the use of multiple measures for each behavioural problem. For instance, SNP heritability estimates are consistent across Conners ADHD and SDQ ADHD measures, in the current study and previous TEDS reports. TEDS also have data from multiple reporters. The other studies relied on maternal report, which may contribute to their higher SNP heritability estimates.

**Sex limitation model-fitting**

The sex limitation model tests for quantitative and qualitative sex differences in the aetiology of phenotypic differences (i.e. testing for differences in the magnitude of genetic and environmental effects, and for the presence of different genetic and environmental influences between the sexes, respectively).

The relative fit of nested sex limitation models was tested with chi-squared difference tests for each of a representative range of 15 behaviour problem phenotypes (see Supplementary table 2). The following models were tested in our analyses: full heterogeneity model (all parameters are allowed to vary across all five zygosity groups), heterogeneity models (fixing either the genetic or shared environmental correlation to their expected value (0.5 or 1 respectively)), and a homogeneity model (equating ACE estimates between the sexes and constraining the DZ opposite-sex genetic correlation to 0.5). A non-significant p-value indicates that the model is consistent with the data, whereas a significant value indicates poor fit.

The results in Supplementary Table 2 suggest that there are no qualitative sex differences for any of the traits, because constraining the correlations to expected values does not significantly reduce model fit. Quantitative sex differences are present for most of the measures (except self-rated depression at 12 and self-rated autism at 16), because equating estimates across sexes does not decrease model fit compared to the heterogeneity model. However, the differences between the heritability estimates for males and females are small (e.g. 38% vs. 41% heritability for self-rated anxiety at 12 in males and females, respectively) with overlapping confidence intervals for nearly all of the behaviour problem measures (see Supplementary Table 3). Therefore, despite being statistically significant, these sex differences are small.

**Supplementary Table 2: comparison sex limitation sub-models**

|  |  | **Model** | **ep** | **minus2LL** | **df** | **AIC** | **diffLL** | **diffdf** | **p** |
| --- | --- | --- | --- | --- | --- | --- | --- | --- | --- |
| 12yr self | SDQ anxiety | Full sex-limited | 9 | 24481.14 | 8859 | 6763.141 | NA | NA | NA |
|  |  | Qualitative (fixed rG) | 8 | 24482.17 | 8860 | 6762.172 | 1.030 | 1 | 0.310 |
|  |  | Qualitative (fixed rC) | 8 | 24482.17 | 8860 | 6762.172 | 0.000 | 1 | 1.000 |
|  |  | Quantitative genetic | 5 | 24718.37 | 8863 | 6992.374 | 236.203 | 3 | 0.000 |
|  | SDQ hyperactivity | Full sex-limited | 9 | 24669.59 | 8924 | 6821.586 | NA | NA | NA |
|  |  | Qualitative (fixed rG) | 8 | 24672.22 | 8925 | 6822.221 | 2.635 | 1 | 0.105 |
|  |  | Qualitative (fixed rC) | 8 | 24672.22 | 8925 | 6822.221 | 0.000 | 1 | 1.000 |
|  |  | Quantitative genetic | 5 | 24814.58 | 8928 | 6958.58 | 142.359 | 3 | 0.000 |
|  | MFQ depression | Full sex-limited | 9 | 24284.98 | 8777 | 6730.981 | NA | NA | NA |
|  |  | Qualitative (fixed rG) | 8 | 24284.98 | 8778 | 6728.981 | 0.000 | 1 | 1.000 |
|  |  | Qualitative (fixed rC) | 8 | 24284.98 | 8778 | 6728.981 | -6.599 | 1 | 1.000 |
|  |  | Quantitative genetic | 5 | 24289.96 | 8781 | 6727.963 | 4.982 | 3 | 0.173 |
| 12yr parent | SDQ composite | Full sex-limited | 9 | 22470.18 | 8845 | 4780.182 | NA | NA | NA |
|  |  | Qualitative (fixed rG) | 8 | 22470.18 | 8846 | 4778.182 | 0.000 | 1 | 1.000 |
|  |  | Qualitative (fixed rC) | 8 | 22470.18 | 8846 | 4778.182 | -436.399 | 1 | 1.000 |
|  |  | Quantitative genetic | 5 | 22619.98 | 8849 | 4921.984 | 149.802 | 3 | 0.000 |
|  | CAST autistic | Full sex-limited | 9 | 23293.37 | 8994 | 5305.37 | NA | NA | NA |
|  |  | Qualitative (fixed rG) | 8 | 23293.37 | 8995 | 5303.37 | 0.000 | 1 | 1.000 |
|  |  | Qualitative (fixed rC) | 8 | 23293.37 | 8995 | 5303.37 | 0.000 | 1 | 1.000 |
|  |  | Quantitative genetic | 5 | 23473.31 | 8998 | 5477.309 | 179.939 | 3 | 0.000 |
|  | APSD psychopathic | Full sex-limited | 9 | 21958.4 | 8849 | 4260.396 | NA | NA | NA |
|  |  | Qualitative (fixed rG) | 8 | 21958.4 | 8850 | 4258.396 | 0.000 | 1 | 1.000 |
|  |  | Qualitative (fixed rC) | 8 | 21958.4 | 8850 | 4258.396 | -603.849 | 1 | 1.000 |
|  |  | Quantitative genetic | 5 | 22056.09 | 8853 | 4350.094 | 97.698 | 3 | 0.000 |
| 12yr teacher | SDQ composite | Full sex-limited | 9 | 19259.27 | 7272 | 4715.275 | NA | NA | NA |
|  |  | Qualitative (fixed rG) | 8 | 19259.95 | 7273 | 4713.95 | 0.675 | 1 | 0.411 |
|  |  | Qualitative (fixed rC) | 8 | 19259.95 | 7273 | 4713.95 | 0.584 | 1 | 0.445 |
|  |  | Quantitative genetic | 5 | 19851.9 | 7276 | 5299.898 | 591.949 | 3 | 0.000 |
|  | CAST autistic | Full sex-limited | 9 | 19677.19 | 7188 | 5301.187 | NA | NA | NA |
|  |  | Qualitative (fixed rG) | 8 | 19678.22 | 7189 | 5300.22 | 1.033 | 1 | 0.309 |
|  |  | Qualitative (fixed rC) | 8 | 19678.22 | 7189 | 5300.22 | 1.017 | 1 | 0.313 |
|  |  | Quantitative genetic | 5 | 19840.23 | 7192 | 5456.229 | 162.009 | 3 | 0.000 |
|  | APSD psychopathic | Full sex-limited | 9 | 18949.35 | 7194 | 4561.347 | NA | NA | NA |
|  |  | Qualitative (fixed rG) | 8 | 18949.35 | 7195 | 4559.347 | 0.000 | 1 | 1.000 |
|  |  | Qualitative (fixed rC) | 8 | 18949.35 | 7195 | 4559.347 | 0.000 | 1 | 1.000 |
|  |  | Quantitative genetic | 5 | 19598.25 | 7198 | 5202.252 | 648.905 | 3 | 0.000 |
| 16yr self | SDQ composite | Full sex-limited | 9 | 21225.11 | 7636 | 5953.111 | NA | NA | NA |
|  |  | Qualitative (fixed rG) | 8 | 21226.11 | 7637 | 5952.112 | 1.001 | 1 | 0.317 |
|  |  | Qualitative (fixed rC) | 8 | 21226.11 | 7637 | 5952.112 | -28.074 | 1 | 1.000 |
|  |  | Quantitative genetic | 5 | 21254.08 | 7640 | 5974.083 | 27.971 | 3 | 0.000 |
|  | AQ autism | Full sex-limited | 9 | 21000.21 | 7600 | 5800.211 | NA | NA | NA |
|  |  | Qualitative (fixed rG) | 8 | 21002.16 | 7601 | 5800.161 | 1.950 | 1 | 0.163 |
|  |  | Qualitative (fixed rC) | 8 | 21002.16 | 7601 | 5800.161 | 0.000 | 1 | 1.000 |
|  |  | Quantitative genetic | 5 | 21005.69 | 7604 | 5797.686 | 3.525 | 3 | 0.318 |
|  | SPEQ hedonia | Full sex-limited | 9 | 20867.8 | 7584 | 5699.795 | NA | NA | NA |
|  |  | Qualitative (fixed rG) | 8 | 20868.02 | 7585 | 5698.017 | 0.222 | 1 | 0.638 |
|  |  | Qualitative (fixed rC) | 8 | 20868.02 | 7585 | 5698.017 | 0.222 | 1 | 0.638 |
|  |  | Quantitative genetic | 5 | 20905.65 | 7588 | 5729.652 | 37.635 | 3 | 0.000 |
| 16yr parent | SDQ composite | Full sex-limited | 9 | 19394 | 7552 | 4290.003 | NA | NA | NA |
|  |  | Qualitative (fixed rG) | 8 | 19394.29 | 7553 | 4288.287 | 0.283 | 1 | 0.594 |
|  |  | Qualitative (fixed rC) | 8 | 19394.29 | 7553 | 4288.287 | 0.283 | 1 | 0.594 |
|  |  | Quantitative genetic | 5 | 19572.32 | 7556 | 4460.323 | 178.036 | 3 | 0.000 |
|  | AQ autism | Full sex-limited | 9 | 18093.26 | 7631 | 2831.263 | NA | NA | NA |
|  |  | Qualitative (fixed rG) | 8 | 18093.26 | 7632 | 2829.263 | 0.000 | 1 | 1.000 |
|  |  | Qualitative (fixed rC) | 8 | 18093.26 | 7632 | 2829.263 | -1108.559 | 1 | 1.000 |
|  |  | Quantitative genetic | 5 | 18153.65 | 7635 | 2883.652 | 60.390 | 3 | 0.000 |
|  | SPEQ neg. symptoms | Full sex-limited | 9 | 18587.53 | 7490 | 3607.528 | NA | NA | NA |
|  |  | Qualitative (fixed rG) | 8 | 18591.41 | 7491 | 3609.412 | 3.884 | 1 | 0.049 |
|  |  | Qualitative (fixed rC) | 8 | 18591.41 | 7491 | 3609.412 | 3.884 | 1 | 0.049 |
|  |  | Quantitative genetic | 5 | 19220.17 | 7494 | 4232.174 | 628.762 | 3 | 0.000 |

**Supplementary Table 3: sex limitation model-fitting results for additive genetic (A), shared environmental (C) and non-shared environmental (E) components of variance, with 95% confidence intervals.**

**Supplementary Table 4: full results for the present study**

**Sensitivity analyses**

We conducted sensitivity analyses to examine the influence of the van der Waerden transformation and removal of outliers on SNP heritability estimates (see Supplementary Figures 1-5). Note that ‘VdW, no outliers’ refers to the procedure followed in our primary analyses, where distributions were age and sex regressed, outliers were removed, and data were quantile normalised with the van der Waerden transformation. The label ‘No VdW, no outliers’ refers to analyses conducted with data that was not quantile normalised (but age and sex regression and outlier removal were performed). ‘VdW, outliers kept’ and ‘VdW, winzorised’ refer to analyses conducted on data that were age- and sex-regressed and transformed, but outliers were simply retained or winzorised (by creating z-scores and then clustering the outliers at z=3.29) rather than removed.

There was no difference in SNP heritability estimates of more than 0.04 between the different sensitivity analyses of 12-year parent-ratings, 12-year teacher-ratings, or 16-year self-ratings of behaviour problems. For self-reported SDQ and MFQ at 12, and parent-rated SDQ at 16, SNP heritability estimates changed by 0.05 for some sensitivity analyses, but we suggest that these differences are not important given that standard errors are almost completely overlapping for all estimates.

The limited distribution of the behaviour problems phenotypes due to positive skew might explain some of the low heritability. A large proportion of the sample score 0-1 on the scales, leaving little information for heritability estimates to work with. Residualising and quantile normalising these values only uses age and sex to give a spread in observations between distribution, but does not add any genetic information for heritability estimation. The combination of the limited distribution of the measures, and the low concordance between raters will substantially reduce power of the heritability studies, which is not accounted for in the power calculations. Future studies could aim to address this issue by developing more normally distributed measures. It would also be interesting to investigate whether the order of data preparation steps affects heritability estimates. For example, transforming residuals might introduce the negative of the effects that were being regressed out.

**Supplementary Figure 1: sensitivity analyses of SNP heritability estimates for 12-year self-reported behaviour problems**


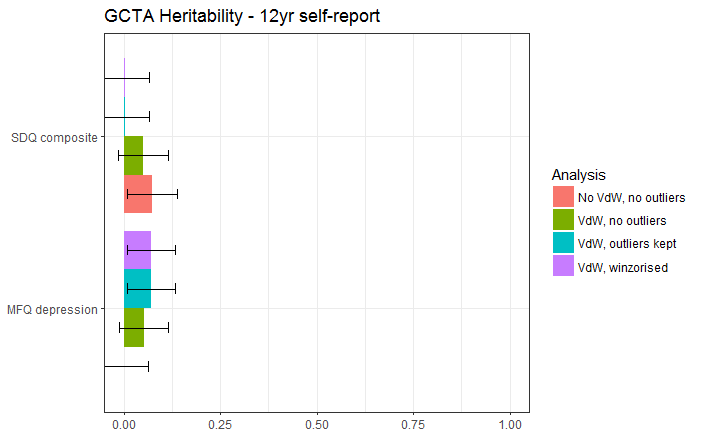


**Supplementary Figure 2: sensitivity analyses of SNP heritability estimates for 12-year parent-rated behaviour problems**


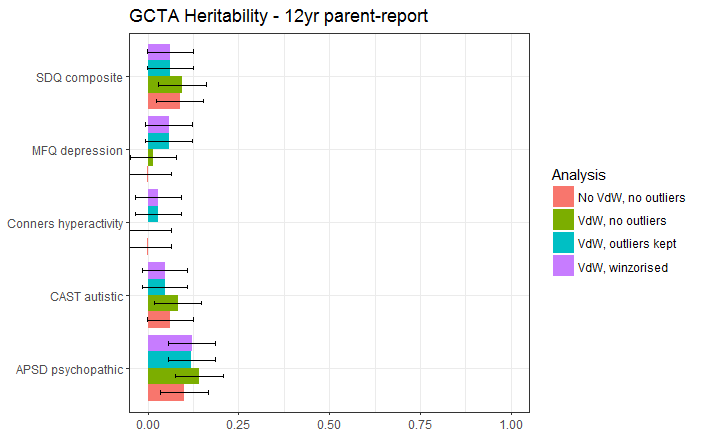


**Supplementary Figure 3: sensitivity analyses of SNP heritability estimates for 12-year teacher-rated behaviour problems**


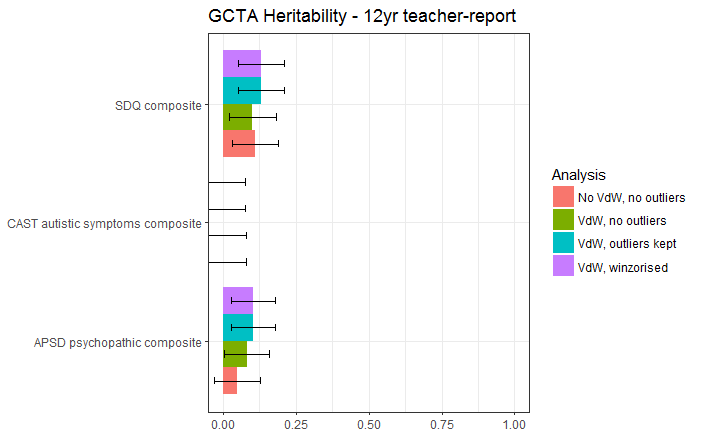


**Supplementary Figure 4: sensitivity analyses of SNP heritability estimates for 16-year self-rated behaviour problems**


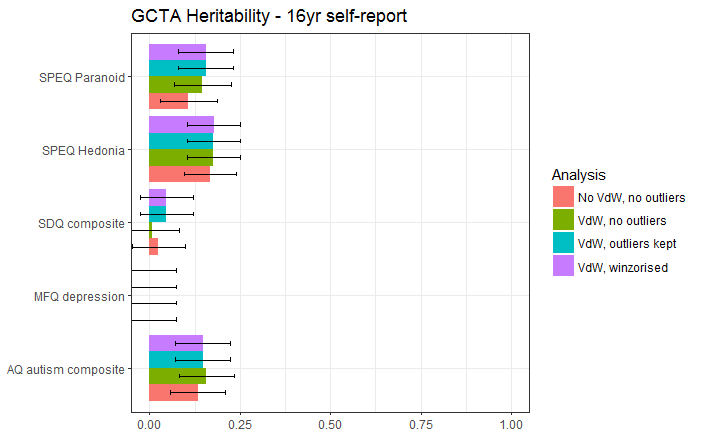


**Supplementary Figure 5: sensitivity analyses of SNP heritability estimates for 16-year parent-rated behaviour problems**


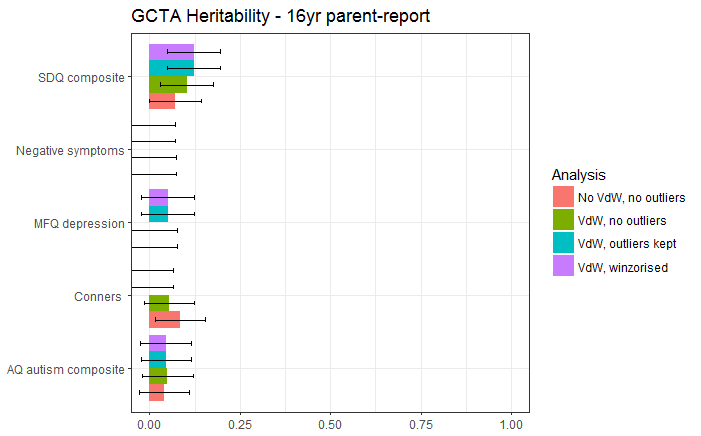


**Supplementary Table 5: ADE model-fitting results.** Note: square brackets contain 95% confidence intervals for the variance component estimates.

| **Measure** | **A** | **D** | **E** |
| --- | --- | --- | --- |
| Self-reported SDQ anxiety 12 | 0.18 [0.03, 0.33] | 0.23 [0.07, 0.40] | 0.58 [0.55, 0.62] |
| Self-reported SDQ hyperactivity at 12 | 0.21 [0.06, 0.36] | 0.26 [0.10, 0.42] | 0.53 [0.50, 0.57] |
| Teacher-rated autism at 12 | 0.51 [0.33, 0.57] | 0.02 [0, 0.20] | 0.47 [0.44, 0.51] |
| Self-reported autism at 16 | 0.31 [0.15, 0.47] | 0.21 [0.05, 0.38] | 0.48 [0.44, 0.51] |
| Self-reported cognitive disorganisation at 16 | 0.41 [0.26, 0.50] | 0.05 [0, 0.22] | 0.53 [0.49, 0.57] |
| Self-reported grandiosity 16 | 0.37 [0.21, 0.49] | 0.09 [0, 0.27] | 0.54 [0.50, 0.58] |
| Self-reported hedonia at 16 | 0.30 [0.14, 0.46] | 0.16 [0, 0.33] | 0.54 [0.50, 0.58] |

For 7 of the 42 behaviour problem variables (the variables in the table), ADE models fit significantly better than ACE models, as indicated by a lower AIC (Akaike’s Information Criterion).

Notably, it is possible that including opposite-sex as well as same-sex DZ twins contributed to this evidence for non-additivity for behaviour problems. If DZ opposite-sex twins correlate less than same-sex DZs, they would reduce the overall DZ correlation. The evidence for sex differences is weak overall, despite the statistical significance of quantitative sex differences for these traits (see Supplementary Tables 2 and 3).

References

1. Benke KS, Nivard MG, Velders FP, Walters RK, Pappa I, Scheet PA, et al. A genome-wide association meta-analysis of preschool internalizing problems. J Am Acad Child Adolesc Psychiatry. 2014 Jun;53(6):667–676.e7.

2. Middeldorp CM, Hammerschlag AR, Ouwens KG, Groen-Blokhuis MM, Pourcain BS, Greven CU, et al. A Genome-Wide Association Meta-Analysis of Attention-Deficit/Hyperactivity Disorder Symptoms in Population-Based Pediatric Cohorts. J Am Acad Child Adolesc Psychiatry. 2016 Oct;55(10):896–905.e6.

3. Neumann A, Pappa I, Lahey BB, Verhulst FC, Medina-Gomez C, Jaddoe VW, et al. Single nucleotide polymorphism heritability of a general psychopathology factor in children. J Am Acad Child Adolesc Psychiatry. 2016 Dec;55(12):1038–1045.e4.

4. Pappa I, Fedko IO, Mileva-Seitz VR, Hottenga J-J, Bakermans-Kranenburg MJ, Bartels M, et al. Single Nucleotide Polymorphism Heritability of Behavior Problems in Childhood: Genome-Wide Complex Trait Analysis. J Am Acad Child Adolesc Psychiatry. 2015 Sep;54(9):737–744.

5. Sallis H, Evans J, Wootton R, Krapohl E, Oldehinkel AJ, Davey Smith G, et al. Genetics of depressive symptoms in adolescence. BMC Psychiatry. 2017 Aug 31;17(1):321.

6. St Pourcain B, Skuse DH, Mandy WP, Wang K, Hakonarson H, Timpson NJ, et al. Variability in the common genetic architecture of social-communication spectrum phenotypes during childhood and adolescence. Mol Autism. 2014 Feb 24;5(1):18.

7. Stergiakouli E, Davey Smith G, Martin J, Skuse DH, Viechtbauer W, Ring SM, et al. Shared genetic influences between dimensional ASD and ADHD symptoms during child and adolescent development. Mol Autism. 2017 Apr 4;8(1):18.

8. Trzaskowski M, Eley TC, Davis OSP, Doherty SJ, Hanscombe KB, Meaburn EL, et al. First genome-wide association study on anxiety-related behaviours in childhood. PLoS ONE. 2013 Apr 2;8(4):e58676.

9. Trzaskowski M, Dale PS, Plomin R. No genetic influence for childhood behavior problems from DNA analysis. J Am Acad Child Adolesc Psychiatry. 2013 Oct;52(10):1048–1056.e3.

10. Sieradzka D, Power RA, Freeman D, Cardno AG, Dudbridge F, Ronald A. Heritability of individual psychotic experiences captured by common genetic variants in a community sample of adolescents. Behav Genet. 2015 Sep;45(5):493–502.

11. Viding E, Price TS, Jaffee SR, Trzaskowski M, Davis OSP, Meaburn EL, et al. Genetics of callous-unemotional behavior in children. PLoS ONE. 2013 Jul 9;8(7):e65789.
